# Do platform recommendation logics shape public engagement with sports health content? 400-video evidence from Douyin and Bilibili

**DOI:** 10.3389/fpubh.2026.1801241

**Published:** 2026-04-16

**Authors:** Zhao Zhang, Bing Shi

**Affiliations:** Shaanxi Normal University, Xi'an, Shaanxi, China

**Keywords:** algorithmic cultural filtering, badminton video, Bilibili, Douyin, sports engagement

## Abstract

**Background:**

Digital video platforms are not neutral conduits; their ranking signals and interfaces shape what becomes visible and valuable. Badminton—balancing spectacle and instruction—offers leverage to compare Douyin's engagement-optimized short video with Bilibili's community- and knowledge-oriented ecology.

**Objective:**

To examine whether engagement patterns differ across platform-native discovery environments and to interpret these differences through an algorithmic cultural filtering lens.

**Methods:**

We analyzed 400 videos sampled in June 2025 (200 per platform) using each platform's native discovery orders; coders recorded content type, creator identity, duration, and public counters (likes, comments, shares, and bookmarks), with excellent inter-coder reliability (κ = 0.94). Because engagement metrics were highly right-skewed, cross-platform differences were first examined using two-tailed Mann–Whitney *U*-tests with effect sizes. We then estimated multivariable linear regression models for log-transformed engagement outcomes, adjusting for platform, content type, video duration, creator identity, and keyword match.

**Conclusion:**

Douyin yields markedly higher instantaneous reactions (likes/comments/shares), whereas favorites/bookmarks converge across platforms; Bilibili hosts longer videos and more instructional content, and creator ecologies diverge (Douyin KOL-led, Bilibili amateur-led). These regularities are consistent with an algorithmic cultural filtering lens, with platform architectures, creator adaptation, and audience preferences jointly shaping visible engagement patterns. Bridge formats (e.g., linking highlights to modular instruction) may connect attention with learning.

## 1 Introduction

Digital video platforms have become pivotal infrastructures for the production, circulation, and interpretation of contemporary sport culture. Building on a decade of scholarship that has moved from describing the uptake of social media in sport toward interrogating its social impacts, metrics, and value creation, current debates emphasize that platforms are not neutral pipes but socio-technical systems that actively shape participation and meaning (1–5). Recommendation algorithms, interface affordances, and business models together filter what is visible and valuable, thereby conditioning how fans, athletes, and creators encounter and co-create sport culture online (6, 7, 24).

Within this literature, cross-platform comparisons remain comparatively scarce—especially outside Western contexts—despite mounting evidence that seemingly similar video platforms cultivate distinct creator ecologies, content grammars, and user engagement repertoires (8). Platform differences matter because they shape how sports are rendered into digital symbols (highlights, breakdowns, challenges), how knowledge is codified (instructional formats), and how community participation unfolds (likes, comments, shares, bookmarks) (4, 9, 10). Moreover, research on online diffusion cautions that observed engagement outcomes reflect complex interactions among algorithmic curation, network structure, and timing—rather than any single determinant (11–14).

We conduct a comparative analysis of badminton content on Douyin and Bilibili—two major Chinese platforms with contrasting logics: Douyin as a short-video, engagement-optimized environment, and Bilibili as a community-oriented, knowledge-oriented site. Badminton is a theoretically informative case: it blends spectacle, skill acquisition, and community identity, allowing us to observe how different platform affordances and incentive systems privilege emotion-driven, visually impactful snippets vs. longer, systematic, instructional content (9, 15–17). We introduce the analytic lens of algorithmic cultural filtering to describe how platform-specific ranking, evaluation, and feedback signals selectively amplify or suppress cultural expressions of sport, thereby shaping expression forms (e.g., highlight vs. instruction), transmission pathways (e.g., instant feedback vs. deep engagement), and value orientations (e.g., attention vs. knowledge).

This concept is related to, but distinct from, existing work on affordances and calculated publics. Affordance-based approaches clarify how platform features enable or constrain possible actions for users and creators, but they do not by themselves specify how ranking, recommendation, and feedback systems selectively elevate some cultural forms over others. By contrast, the notion of calculated publics highlights how algorithmic systems render publics legible to themselves through metrics, visibility, and ranking. Our use of algorithmic cultural filtering shifts the analytical focus from action possibilities or public legibility alone to the selective amplification and attenuation of cultural expressions, transmission pathways, and value orientations across platform-specific discovery environments. Its added theoretical value lies in linking platform infrastructures, creator adaptation, and user engagement within a comparative framework that explains why the same sport is formatted and valued differently across platforms.

Empirically, we analyze 400 badminton videos sampled in June 2025 (200 per platform) using a standardized content-analytic protocol and cross-platform-appropriate ranking criteria, and we model user interactions (likes, comments, shares, bookmarks) alongside content and creator attributes. This design enables us to compare platform–content–user triadic dynamics and quantify differences in engagement patterns while acknowledging ecological validity across the two platform environments.

The study makes four contributions. First, theoretically, we propose algorithmic cultural filtering as a comparative meso-level lens that complements affordance-based and calculated-publics approaches by focusing on how platform-specific ranking, feedback, and evaluation systems selectively amplify different cultural forms, transmission pathways, and value orientations. Second, methodologically, we present a cross-platform comparative design that respects platform-specific evaluation signals and complements non-parametric testing with effect sizes, consistent with diffusion and engagement research that cautions against over-interpreting single metrics. Third, empirically, we extend sport communication research beyond Western platforms by analyzing two highly consequential Chinese ecosystems, detailing how creator types (official, KOL, grassroots, player) and content formats map onto platform incentives. Fourth, practically, we derive actionable implications for platform operators (metric portfolios that balance instant feedback with depth signals), creators (format–platform fit), and sport organizations (digital transformation strategies that leverage community learning and sustained value rather than short-term attention).

Conceptually, our framework foregrounds two distinct but coexisting logics: the accumulation of emotional capital through rapid, low-cost signals (likes, brief comments, quick shares) and the accumulation of knowledge capital through time-intensive signals (completion, bookmarking, in-depth discussion). We situate these logics within classic theories of affordances and capital to explain why certain sport-content grammars thrive on one platform yet plateau on another (13, 18–20). In sum, by explicating how platform differentiation reconfigures the cultural transmission of a global sport, the study advances comparative platform scholarship in sport and offers a transferable lens for examining other technique-rich sports and video-centric ecosystems.

## 2 Materials and methods

### 2.1 Research framework

We develop a triadic interaction framework—platform algorithm–creator–user—to structure the comparative analysis of sports-related video circulation across platforms. The framework integrates algorithmic curation (24), platformization (6), and connectivity culture (5) to link technical architectures with production incentives and participation patterns. As the core analytic lens, algorithmic cultural filtering is treated not as a unidirectional cause but as a technology-mediated process in which ranking, recommendation, and evaluation systems selectively surface or suppress cultural content through platform-specific parameters and signals. This formulation enables examination of how platform logics condition content visibility and meaning-making while remaining agnostic about technological determinism and attentive to creators' practices and users' routines.

Operationally, we analyze a stratified sample of 400 badminton videos collected in June 2025 (200 per platform) and code video-, creator-, and context-level attributes with a standardized protocol adapted to each platform's affordances. Content variables include format (e.g., highlight, instructional, match excerpt), visual/editing features, and topical tags; creator variables capture organizational status (official, KOL, grassroots, player) and production cadence; contextual variables include title/hashtag metadata and posting time. Engagement is measured using platform-available indicators (likes, comments, shares, bookmarks/favorites, and, where available, dwell-time proxies). Because platform interfaces do not provide reliable viewer-level demographic or skill-profile information for sampled videos, engagement is operationalized here as aggregate platform-visible interaction rather than audience-segment-specific response. Cross-platform comparability is addressed by aligning construct definitions and normalizing metrics where necessary. Analysis proceeded through descriptive statistics, non-parametric comparisons with effect sizes, and multivariable linear regression models for log-transformed engagement outcomes, including platform indicators and content- and creator-level covariates. Inferences about platform-specific filtering are drawn from observable regularities in these signals and documented affordances, providing an empirically grounded account of how platform differentiation structures badminton-related cultural transmission.

### 2.2 Data collection

Data were collected in June 2025 using a platform-sensitive keyword sampling protocol designed to preserve each site's native ranking logic. The June 2025 collection window should also be read against the contemporaneous badminton calendar: it followed the 2025 Sudirman Cup (27 April−4 May) and overlapped with the Singapore Open (27 May−1 June) and Indonesia Open (3–8 June). These event cycles may have temporarily elevated the visibility of match highlights, commentary, instructional analysis, or challenge-related content during the sampling period. The sampling frame comprised 400 videos, with 200 drawn from Douyin and 200 from Bilibili. To capture the progression from consumption to learning to participation, we queried five theoretically motivated keywords—badminton, badminton instruction, badminton matches, badminton highlights, and badminton challenges—grounded in uses-and-gratifications theory and the consumer decision journey model (21, 22). Following guidance on algorithmic sorting logic and the principle of platform specificity in digital methods (23, 24), we retained each platform's native ranking signals when drawing the sample. On Douyin, we used the “most likes” order and collected the top 40 results for each keyword (5 × 40 = 200), reflecting that platform's emphasis on instantaneous feedback metrics. On Bilibili, we combined two discovery orders— “most views” and “most bookmarks”—and collected the top 20 results under each order for every keyword (2 × 20 × 5 = 200), aligning with Bilibili's stronger orientation toward depth signals and sustained engagement. This differentiated protocol avoids distortions that can arise from imposing a uniform ranking criterion across platforms and enhances representativeness within each platform's algorithmic ecology. Although we did not stratify by upload date, the unified collection window supports contemporaneous comparison; because the study focuses on relative cross-platform differences, effect-size estimation is subsequently used to reduce sensitivity to residual temporal variation. At the same time, this choice means that the sampled corpus is partly structured by platform-specific ranking criteria themselves. Accordingly, the observed cross-platform differences should be interpreted as differences in content as surfaced through each platform's native discovery system, rather than as differences derived from a neutral or fully equivalent sampling frame.

### 2.3 Theoretical justification

Our differentiated sampling strategy follows the platform localization principle grounded in affordances theory: the functional characteristics of a technical system shape the action possibilities available to users and creators (16). In practice, Douyin's ranking logic foregrounds instantaneous feedback (e.g., “most likes”), whereas Bilibili emphasizes depth-oriented signals (e.g., “most views,” “most bookmarks”). Publicly available platform disclosures also suggest that the two systems differ beyond the visible ranking labels used in sampling. Douyin's disclosed recommendation logic relies on behavioral prediction over signals such as click, watch time, likes, comments, shares, reposts, and negative feedback, followed by ranking, diversification, and intervention; more recent transparency materials further indicate a multi-objective framework that incorporates favorites, revisit behavior, follow-through, and search-linked long-term value signals. By contrast, Bilibili's disclosed homepage recommendation logic combines user features, candidate-pool content, and contextual factors, and fuses positive and negative behavioral signals including plays, likes, coins, favorites, follows, shares, dislikes, and “not interested,” while also considering diversity and content quality in final ranking. We therefore treat the platform contrast in this study not as a simple difference in surface metrics, but as a difference in documented recommendation architectures that weight immediacy, retention, and community endorsement in different ways.

Imposing a uniform ordering criterion across these environments would misrepresent their native ecologies and introduce systematic sampling bias. We therefore adopt ecological adaptive sampling, recognizing that cross-platform comparison should reveal—not erase—environmental differences. Consistent with the platformization literature, platform logics and content formats co-adapt over time; preserving platform-native ranking signals allows the sample to capture this co-evolutionary process rather than an artifact of externally enforced standardization (6).

A further rationale is comparative validity. By drawing samples that are representative within each platform's own algorithmic context, we compare “badminton content as surfaced by Douyin” with “badminton content as surfaced by Bilibili,” thereby strengthening construct validity and the practical interpretability of cross-platform contrasts. To safeguard sample quality, we applied uniform exclusion rules across platforms: videos unrelated to badminton were removed; clips shorter than 10 s were excluded to ensure a meaningful unit of analysis; duplicate items appearing under multiple keywords or orders were deduplicated; and the retained items were balanced across the five keywords (badminton, badminton instruction, badminton matches, badminton highlights, badminton challenges) to maintain comparable topical coverage across platforms.

### 2.4 Core variables and coding

The core variable definitions and coding rules are summarized in Table 1. This study operationalizes three families of variables—basic descriptors, dissemination behaviors, and content semantics—to enable cross-platform comparison while preserving each platform's native metrics. All public counters were recorded as displayed by the platforms; where indicated, values are stored in units of 10,000 (wan) to match interface conventions. When a video contains multiple elements (e.g., highlight plus instruction), coding follows the dominant functional purpose. Creator identity is determined from verified status, follower size, and demonstrated expertise; “official” denotes organizations or media outlets, “KOL” denotes influential professional creators (typically ≥100k followers), “athlete” denotes professional or semi-professional players, and “amateur” denotes general enthusiasts.

**Table 1:** Core variable definitions and coding rules.

Variable category	Variable name	Variable type	Coding rules
Basic information	Platform source	Categorical variable	1 = Douyin, 2 = Bilibili
Basic information	Video DURATION	Continuous variable	Actual duration values (unit: seconds)
Basic information	Publication time	Categorical variable	Grouped by publication date
Basic information	Search keywords	Categorical variable	1 = badminton, 2 = badminton instruction, 3 = badminton matches, 4 = badminton highlights, 5 = badminton challenges
Transmission behavior	View count	Continuous variable	Actual values (Unit: 10,000, Bilibili only)
Transmission behavior	Like count	Continuous variable	Actual values (Unit: 10,000)
Transmission behavior	Comment count	Continuous variable	Actual values (Unit: 10,000)
Transmission behavior	Share count	Continuous variable	Actual values (Unit: 10,000)
Transmission behavior	Bookmark count	Continuous variable	Actual values (Unit: 10,000)
Transmission behavior	Like rate	Continuous variable	Like count/view count × 100% (Bilibili only)
Transmission behavior	Bookmark rate	Continuous variable	Bookmark count/view count × 100% (Bilibili only)
Transmission behavior	Danmu (barrage) density	Continuous variable	Length-adjusted frequency of danmu (time-synchronized on-screen comments), calculated as danmu count divided by video duration (Bilibili only).
Transmission behavior	Interaction index	Continuous variable	(Like count + comment count × 2 + share count × 3)/1,000
Content semantics	Content type	Categorical variable	1 = news/events (official reports), 2 = highlights (spectacular moments), 3 = science/analysis (rule explanations), 4 = instruction (technical guidance), 5 = entertainment (humorous creativity), 6 = live matches (complete games), 7 = challenges (interactive tests), 8 = match compilations (edited collections)
Content semantics	Creator subject	Categorical variable	1 = official (authoritative publishers), 2 = KOL (professional bloggers), 3 = grassroots (ordinary enthusiasts), 4 = players (professional athletes)
Content semantics	Keyword matching	Categorical variable	1 = match, 2 = no match

On Bilibili, danmu (barrage) refers to time-synchronized viewer comments displayed directly on the video screen during playback. In this study, danmu density was calculated as danmu count divided by video duration and was treated as a platform-specific descriptive indicator rather than a primary cross-platform outcome.

Rationale for the engagement index. We constructed this interaction index as an exploratory composite indicator rather than as a definitive measure of engagement value. The weighting scheme of 1:2:3 for likes, comments, and shares was specified *a priori* to reflect an ordinal increase in participation effort and outward social exposure: likes represent low-cost affective reactions, comments require greater time and cognitive involvement, and shares involve redistribution to others. This ordering is conceptually consistent with the COBRAs framework, which distinguishes progressively more active forms of online engagement (20). Because no universally accepted weighting standard exists for these actions, the index is used here as a supplementary summary measure, while the main inferential analyses are reported separately for likes, comments, shares, and favorites/bookmarks.

### 2.5 Statistical analysis

Data were gathered through a multi-stage, programmatic workflow. First, videos were retrieved via each platform's native search using the pre-defined keywords and the platform-specific ranking orders described above. Second, the raw corpus was systematically screened and cleaned to remove items unrelated to badminton, clips shorter than 10 s, and duplicates across keywords or orders; retained items were then balanced across the five keywords. Third, trained coders extracted information with a structured form aligned to the codebook for variable definitions and decision rules. To ensure coding reliability, we implemented a multi-step protocol: a detailed coding manual with explicit category criteria; a pilot of 50 videos to refine rules; and a formal reliability study in which 80 videos (20% of the corpus), randomly sampled from both platforms, were double-coded and re-coded after a 2-week interval. Agreement was assessed with Cohen's κ, yielding mean κ = 0.94 for Douyin and κ = 0.94 for Bilibili, indicating excellent reliability (κ > 0.80). Because most variables are objective counters (e.g., duration, likes), subjective judgements were limited to content type and creator identity and were anchored to verifiable cues (e.g., verified status, follower count, primary functional purpose), which supported consistency and reproducibility.

Prior to inferential analyses, distributional diagnostics were conducted. Shapiro–Wilk tests indicated significant right-skew for key engagement metrics (e.g., likes, comments, and shares; *p* < 0.001), violating normality assumptions for raw outcomes. Therefore, unadjusted cross-platform comparisons were conducted using two-tailed Mann–Whitney *U*-tests with effect sizes (*r*). For adjusted analyses, we estimated four multivariable linear regression models using log-transformed engagement outcomes [ln (y + 1)] for likes, comments, favorites/bookmarks, and shares. This transformation was applied to reduce skewness and lessen the influence of extreme values, thereby improving the suitability of the outcomes for linear modeling. Covariates included platform type, content type, video duration, creator type, and keyword match. All analyses were conducted in SPSS 27.0, and all tests were two-tailed with α = 0.05.

## 3 Result

### 3.1 Baseline characteristics

(1) Platform-level characteristics and technical differences

Table 2 summarizes baseline attributes for the 400 sampled badminton videos (200 per platform). Clear cross-platform differences emerge in duration profiles, keyword match performance, and ranking architectures. Bilibili exhibits substantially longer videos on average (262.3 s vs. 141.3 s for Douyin), a wider upper tail (maximum 2,736 s vs. 1,418 s), and a greater share of long-form items (>10 min: 27 vs. 10). Douyin, by contrast, concentrates more heavily in the short-form range (< 1 min: 89 vs. 83). Keyword match rates also differ: Douyin shows higher match precision (84.0%) than Bilibili (63.5%). Both platforms are dominated by highlight-style content; however, the least represented categories diverge (compilations on Douyin vs. full-match recordings on Bilibili). Creator ecosystems differ as well: Douyin is led by KOLs with amateurs secondary, whereas Bilibili is led by amateurs with KOLs secondary. Finally, the platforms rely on distinct discovery logics in the sampling frame: Douyin rankings are likes-centric, while Bilibili rankings combine views and bookmarks.

**Table 2:** Cross-platform baseline characteristics of sampled badminton videos (*N* = 400).

Dimension	Indicator	Douyin (*n* = 200)	Bilibili (*n* = 200)
Duration profile	Mean duration (s)	141.3	262.3
	Minimum (s)	10	15
	Maximum (s)	1,418	2,736
	< 1 min (count)	89	83
	1–10 min (count)	101	90
	>10 min (count)	10	27
Keyword precision	Match rate (%)	84.0	63.5
	Matched (count)	168	127
	Not matched (count)	32	73
Content distribution	Most frequent type	Highlights	Highlights
	Least frequent type	Compilations (montage)	Full match (live/replay)
Creator ecosystem	Dominant group	KOL	Amateur
	Secondary group	Amateur	KOL
Sampling/ranking logic	Primary ordering	Most likes	Most views/most bookmarks

Durations are reported in seconds. Keyword match rate reflects whether the retrieved item's primary topic aligns with the queried term. Content-type and creator categories follow the operational definitions in Section 2.4. Discovery logic reflects the platform-native ordering used during sampling (Section 2.2).

(2) Distribution of content types and creator identities

**Figure 1:**
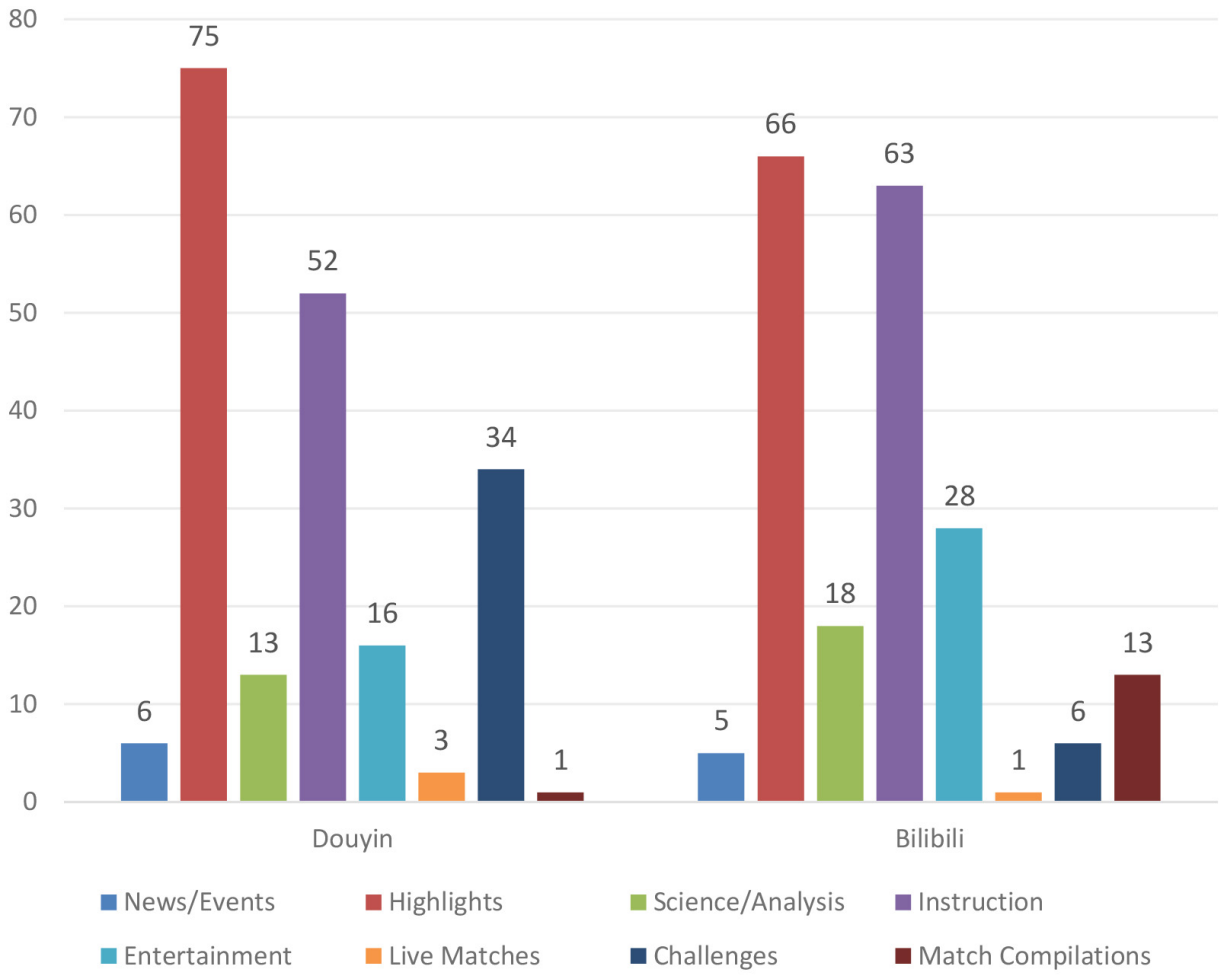
Distribution of content types across platforms (unit: videos).

Across the eight content categories, the two platforms diverge markedly in composition and emphasis. Both are dominated by highlights, with a stronger concentration on Douyin (75 vs. 66 on Bilibili). Instructional content is comparatively more prevalent on Bilibili (63 vs. 52 on Douyin) and is longer on average (mean 428.5 s). Differences are most pronounced in several categories: Douyin far exceeds Bilibili in explanatory/analysis items (34 vs. 6), whereas Bilibili leads in challenges (28 vs. 16), entertainment (18 vs. 13), and compilations (13 vs. 1). News/event reports (6 on Douyin; 5 on Bilibili) and full-match recordings (3 vs. 1) are minority categories on both platforms. Overall, Douyin exhibits a “highlights + explanatory/analysis” concentration, while Bilibili presents a more diversified mix centered on instructional formats (see Figure 1).

Creator composition also differs systematically between platforms. Douyin is KOL-led (65%), with amateurs secondary (24%), whereas Bilibili reverses this pattern, with amateurs leading (55%) and KOLs secondary (40%). Official accounts constitute a small minority on both platforms (7% on Douyin; 2% on Bilibili), and athlete accounts are least common (4 vs. 3%). A complementary analysis of title keywords shows that Douyin frequently deploys emotion-triggering terms (e.g., “highlight,” “challenge,” “funny,” “intense”), aligning with its concentration in highlights, while Bilibili titles more often include knowledge-oriented terms (e.g., “instruction,” “technique,” “complete,” “analysis,” “tips”), mirroring the platform's relative strength in instructional content (see Table 3).

**Table 3:** Distribution of creator identities by platform (*N* = 400).

Creator identity	Douyin (*n* = 200) – count	Douyin (%)	Bilibili (*n* = 200) – count	Bilibili (%)
Official	14	7	4	2
KOL	130	65	80	40
Amateur	48	24	110	55
Athlete	8	4	6	3

Content categories follow the operational definitions in Section 2.4. Percentages are computed within platform. Figure 1 presents category counts; instructional-video mean duration on Bilibili is 428.5 s.

(3) Association between creator ecology and content duration

A stratified summary of creator identity × content-type preferences indicates systematic cross-platform differences in duration profiles (Table 4). Across all creator groups, Bilibili videos are longer than their Douyin counterparts (e.g., official: 235.5 s vs. 32.6 s, ≈7.2 × ; KOL: 377.8 s vs. 162.6 s, ≈2.3 × ; amateur: 172.4 s vs. 116.2 s, ≈1.5 × ; athlete: 387.8 s vs. 137.5 s, ≈2.8 × ). On Douyin, official, KOL, and amateur accounts most frequently produce highlights with sub−3-min averages (39.0–132.0 s), while athlete accounts most often post instruction (161.4 s). On Bilibili, a clearer division of labor emerges: KOLs and athletes concentrate on instruction, with mean durations between 521.0 s and 641.0 s, whereas official and amateur accounts most often publish highlights (211.7–247.5 s). These patterns are consistent with a short-form, visually oriented production logic on Douyin and a longer-form, knowledge-oriented orientation on Bilibili.

**Table 4:** Creator identity, dominant content type, and duration by platform.

Platform	Creator identity	Overall mean duration (s)	Most frequent content type	Mean duration of that type (s)
Douyin	Official	32.6	Highlights	39.0
Douyin	KOL	162.6	Highlights	132.0
Douyin	Amateur	116.2	Highlights	83.6
Douyin	Athlete	137.5	Instruction	161.4
Bilibili	Official	235.5	Highlights	247.5
Bilibili	KOL	377.8	Instruction	521.0
Bilibili	Amateur	172.4	Highlights	211.7
Bilibili	Athlete	387.8	Instruction	641.0

“Most frequent content type” refers to the modal category posted by that creator group on the platform. Durations are in seconds.

Content-type–specific duration profiles reinforce this divergence (Table 5). Bilibili exhibits longer averages in most categories—particularly instruction (428.5 s vs. 194.7 s on Douyin, ≈2.2 × ) and news/events (102.4 s vs. 23.3 s, ≈ 4.4 × ). Full-match recordings show the greatest spread (Bilibili mean 2,736 s vs. Douyin 803 s), reflecting a much wider tolerance for long-form material on Bilibili. Entertainment is comparable across platforms (≈30 s), while Douyin is slightly longer for challenges and compilations. Creator participation by content type also diverges: on Douyin, KOLs dominate most categories (highlights, explanatory/analysis, instruction, full match, challenge), whereas on Bilibili amateurs lead in news/events, highlights, and full match, and KOLs concentrate in instruction and explanatory/analysis.

**Table 5:** Content-type duration profile, main producing creator, and extreme values (platform comparison).

Content type	Douyin mean (s)	Main producing creator (Douyin)	Min/max (s) (Douyin)	Bilibili mean (s)	Main producing creator (Bilibili)	Min/max (s) (Bilibili)
News/events	23.3	Official	16/31	102.4	Amateur	22/287
Highlights	106.3	KOL	11/425	178.5	Amateur	15/2,171
Explanatory/analysis	208.2	KOL	46/886	340.3	KOL	18/1,458
Instruction	194.7	KOL	12/999	428.5	KOL	13/2,093
Entertainment	29.6	Amateur	10/87	30.0	Amateur	10/160
Full match (live/replay)	803.0	KOL	255/1,418	2,736.0	Amateur	2,736
Challenge	121.3	KOL	14/272	77.7	Amateur	17/180
Compilation (montage)	324.0	Amateur	324/324	231.4	Amateur	64/553

Means and extrema are computed within platform and category. “Main producing creator” indicates the creator type contributing the largest share of videos in that category on the platform. Durations are in seconds.

### 3.2 Platform differences in transmission effects

(1) Cross-platform differences in core engagement indicators

Building on the platform-level contrasts documented above, we compared four core engagement metrics—likes, comments, favorites/bookmarks, and shares—using Mann–Whitney *U*-tests with effect sizes reported as $=|Z|/\sqrt{N}$ . Table 6 summarizes medians and interquartile ranges (IQRs) for each platform (values recorded in units of 10,000 to match interface conventions). Three indicators show large, highly significant cross-platform gaps: likes (Douyin median 16.25 vs. Bilibili 3.00; *Z* = −10.64, *p* < 0.001, *r* = 0.53), comments (0.37 vs. 0.03; *Z* = −13.22, *p* < 0.001, *r* = 0.66), and shares (1.90 vs. 0.16; *Z* = −12.35, *p* < 0.001, *r* = 0.62). Favorites/bookmarks do not differ significantly (*Z* = −1.32, *p* = 0.186, *r* = 0.07). These results indicate distinct participation modes: Douyin concentrates high-frequency, low-cost interactions consistent with an “immediate feedback” logic, whereas Bilibili's depth orientation is not captured by instantaneous counters; the convergence in favorites/bookmarks suggests a shared layer of value recognition across both communities.

**Table 6:** Cross-platform comparison of dissemination metrics [median (IQR)].

Metric	Douyin median [IQR]	Bilibili median [IQR]	*Z*	*p*	*r*
Likes	16.25 [7.43–35.18]	3.00 [1.20–8.80]	−10.64	^***^	0.53
Comments	0.37 [0.13–1.10]	0.03 [0.02–0.06]	−13.22	^***^	0.66
Favorites/Bookmarks	0.99 [0.46–3.00]	0.79 [0.37–3.00]	−1.32	0.186	0.07
Shares	1.90 [0.51–5.18]	0.16 [0.04–0.44]	−12.35	^***^	0.62

Mann–Whitney U-tests; effect size. Two-tailed α = 0.05.

^***^p < 0.001. Counts are stored in units of 10,000.

(2) Platform-specific adaptability by content type

To examine how content types align with platform logics, we compared the engagement index (defined in Section 2.4) across eight badminton categories using Mann–Whitney *U*-tests with effect sizes $r\;=\;|Z|/\sqrt{N}$. As shown in Table 7, four categories display significant cross-platform separation. Highlights show the largest difference (Douyin median 0.03 vs. Bilibili 0.00; *Z* = −7.38, *p* < 0.001, *r* = 0.37, medium-to-large), followed by instruction (*Z* = −6.48, *p* < 0.001, *r* = 0.32, medium) and challenge (*Z* = −3.45, *p* < 0.001, *r* = 0.17, small). Explanatory/analysis also differs (*Z* = −2.76, *p* < 0.01, *r* = 0.14, small). By contrast, news/events (*r* = 0.06) and entertainment (*r* = 0.07) show negligible effects, indicating broadly similar performance across platforms. Full-match and compilation categories could not be validly compared due to sparse or absent observations on one platform. Overall, Douyin exhibits an advantage for visually impactful and interaction-prone formats (highlights, instruction, explanatory/analysis, challenge), whereas news and entertainment appear platform-neutral, consistent with cross-platform uptake of timely and light-weight content.

**Table 7:** Platform adaptability by content type [engagement index; median (IQR)].

Content type	Douyin engagement index [IQR]	Bilibili engagement index [IQR]	*Z*	*P*	*r*
News/events	0.03 [0.01–0.15]	0.02 [0.00–0.02]	−1.10	0.273	0.06
Highlights	0.03 [0.01–0.07]	0.00 [0.00–0.01]	−7.38	^***^	0.37
Explanatory/analysis	0.04 [0.00–0.06]	0.00 [0.00–0.01]	−2.76	^**^	0.14
Instruction	0.02 [0.01–0.04]	0.00 [0.00–0.01]	−6.48	^***^	0.32
Entertainment	0.02 [0.00–0.09]	0.01 [0.00–0.02]	−1.34	0.180	0.07
Full match (live/replay)	0.016 [0.00–]	N/A	N/A	N/A	N/A
Challenge	0.03 [0.02–0.05]	0.01 [0.00–0.01]	−3.45	^***^	0.17
Compilation (montage)	N/A	0.00 [0.00–0.01]	N/A	N/A	N/A

Mann–Whitney U-tests; two-tailed α = 0.05; effect size. Significance codes:

^*^p < 0.05,

^**^p < 0.01,

^***^p < 0.001.

The engagement index is defined as (likes + 2 × comments + 3 × shares)/ 1,000 (Section 2.4). “N/A” indicates insufficient or zero observations on one platform, precluding statistical comparison.

(3) Creator-type differences in platform dissemination efficiency

To assess how creator identity interacts with platform characteristics, we compared the engagement index across four creator types—official organizations, KOLs, amateurs, and athletes—using Mann–Whitney *U*-tests with effect sizes $r\;=\;|Z|/\sqrt{N}$. Results (Table 8) indicate a clear advantage for individual creators on Douyin. KOLs achieve substantially higher engagement on Douyin than on Bilibili (*Z* = −9.04, *p* < 0.001, *r* = 0.45; medium-to-large), and amateurs show a similar Douyin advantage (*Z* = −5.30, *p* < 0.001, *r* = 0.27; small-to-medium). By contrast, official accounts (*Z* = −1.91, *p* = 0.056, *r* = 0.10) and athletes (*Z* = −1.16, *p* = 0.245, *r* = 0.06) do not differ significantly across platforms. Taken together, the pattern suggests that Douyin's interaction dynamics disproportionately benefit personal creators (KOLs and amateurs), whereas performance for institutional and athlete accounts remains comparatively platform-neutral.

**Table 8:** Creator-type performance across platforms (engagement index; median [IQR]).

Creator type	Douyin engagement index [IQR]	Bilibili engagement index [IQR]	*Z*	*p*	*r*
Official	0.03 [0.02–0.08]	0.01 [0.00–0.02]	−1.91	0.056	0.10
KOL	0.03 [0.01–0.05]	0.00 [0.00–0.01]	−9.04	^***^	0.45
Amateur	0.02 [0.01–0.06]	0.00 [0.00–0.01]	−5.30	^***^	0.27
Athlete	0.02 [0.01–0.04]	0.01 [0.01–0.02]	−1.16	0.245	0.06

Mann–Whitney U-tests; two-tailed α = 0.05. Effect size $r\;=\;|Z\;|/\sqrt{N}$.

Significance codes:

^***^p < 0.001. The engagement index is defined in Section 2.4 as (likes + 2 × comments + 3 × shares)/1,000.

### 3.3 Determinants of dissemination

(1) Multivariable linear regression results

To identify factors associated with dissemination outcomes, we estimated four multivariable linear regression models using log-transformed engagement outcomes with likes, comments, favorites/bookmarks, and shares as dependent variables. Covariates included platform type, content type, video duration, creator type, and keyword match (see Section 2.4 for coding). Table 9 reports regression coefficients (β) and two-tailed *p*-values. The platform variable shows the most consistent association with engagement: it is negatively related to likes (β = −0.30, *p* < 0.001), comments (β = −0.24, *p* < 0.001), and shares (β = −0.24, *p* < 0.001), indicating lower values on the platform coded with the higher label (Bilibili) relative to the reference (Douyin). Content type is negatively associated with likes (β = −0.03, *p* < 0.001) but not with the other outcomes. Keyword match exhibits a specific association with favorites/bookmarks (β = −0.14, *p* < 0.01) and is non-significant elsewhere. Video duration and creator type do not reach significance in any model. Model fit is modest (adjusted *R*^2^ between 0.01 and 0.05), which is expected for platform-mediated engagement measures that are shaped by many unobserved influences, including posting time, prior follower base, recommendation dynamics, and rapidly changing audience attention. These models are therefore used to identify relative associations among observed variables rather than to provide high-precision prediction of engagement outcomes.

**Table 9:** Multivariable linear regression analysis of factors associated with log-transformed dissemination outcomes.

Predictor	Likes β	Likes *p*	Comments β	Comments *p*	Favorites β	Favorites *p*	Shares β	Shares *p*
Platform type	−0.30	^***^	−0.24	^***^	−0.09	0.117	−0.24	^***^
Content type	−0.03	^***^	−0.02	0.683	−0.00	0.980	−0.08	0.092
Video duration	−0.01	0.774	0.08	0.129	0.00	0.989	0.08	0.100
Creator type	−0.07	0.142	−0.04	0.486	0.04	0.441	−0.03	0.540
Keyword match	−0.05	0.362	0.05	0.285	−0.14	^**^	0.02	0.686
*R* ^2^	0.11		0.06		0.03		0.06	
Adjusted *R*^2^	0.01		0.05		0.02		0.05	
*F* statistic	9.82		4.77		2.72		5.19	

Two-tailed tests; significance codes:

^*^p < 0.05,

^**^p < 0.01,

^***^p < 0.001.

“Platform type” is coded per Section 2.4 (higher value = Bilibili). Other categorical predictors are entered via indicator coding with reference categories defined in the codebook.

(2) Visualizing relative contributions of predictors

**Figure 2:**
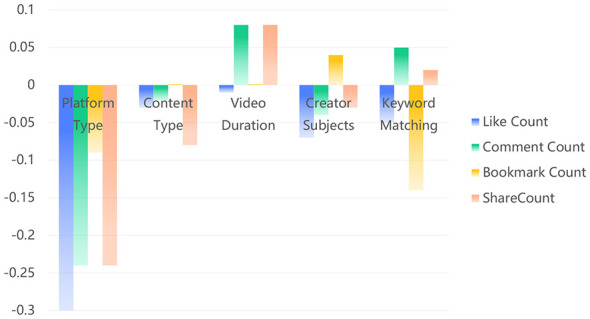
Standardized coefficients (β) and signs for predictors of likes, comments, favorites/bookmarks, and shares.

To complement the regression estimates, we visualized standardized coefficients (β) for each outcome—likes, comments, favorites/bookmarks, and shares—thereby conveying the relative contribution of each predictor on a common scale (Figure 2). Bars represent standardized β from the models in Table 9 (platform type, content type, video duration, creator type, keyword match), with negative values indicating lower expected outcomes as the predictor increases (for categorical variables, relative to the reference category defined in Section 2.4). For interpretability, platform type is coded such that higher values correspond to Bilibili; thus, negative coefficients indicate lower engagement on Bilibili than on Douyin, holding other variables constant.

The visualization underscores the dominant role of platform differences. Platform type exhibits the largest negative coefficients across three outcomes—likes (β=-0.30), comments (β = −0.24), and shares (β = −0.24)—visibly exceeding the magnitudes of other predictors. In contrast, content type, video duration, and creator type cluster near the zero line across outcomes, indicating limited incremental contributions in these linear specifications. A notable exception is keyword match for favorites/bookmarks (β = −0.14), which appears as a clearly negative bar under that outcome and aligns with the regression results, suggesting that keyword alignment is associated with lower bookmarking after adjusting for other factors. Taken together, the coefficient profiles visualize a consistent pattern: platform architecture and associated engagement logics are the factors most strongly associated with the observed dissemination differences, while other variables play more limited roles within the present modeling framework.

## 4 Discussion

This study shows that Douyin and Bilibili organize badminton culture through distinct—but partially overlapping—logics of visibility, participation, and value recognition. Relative to Bilibili, Douyin concentrates short-form, emotionally salient formats and elicits substantially higher volumes of low-cost, instantaneous reactions; in our sample the median like count was 5.4 × higher (16.25 vs. 3.00), the median comment count 12 × higher (0.37 vs. 0.03), and shares were also markedly higher, whereas favorites/bookmarks converged across platforms (0.99 vs. 0.79, *p* = 0.186). Bilibili hosted longer videos on average (262.3 s vs. 141.3 s), a heavier upper tail (>10 min: 27 vs. 10), and a comparative concentration of instructional material (mean 428.5 s vs. 194.7 s), with amateurs leading production (55%) compared with Douyin's KOL-led ecology (65%). Together these regularities indicate differentiated “fit” between content grammars and platform evaluation signals.

We conceptualize these results through *algorithmic cultural filtering*: platform-specific ranking, evaluation, and feedback systems selectively amplify or suppress cultural expressions (e.g., highlights vs. instruction), transmission pathways (instant feedback vs. deep engagement), and value orientations (attention vs. knowledge). The evidence supports this lens while cautioning against technological determinism. First, the strongest platform effects appear on instantaneous counters (likes, comments, shares; *r* = 0.53–0.66), which are precisely the signals that Douyin's engagement-optimized environment foregrounds. Second, a shared stratum of “longer-term value” persists across platforms, visible in the indistinguishable favorites/bookmarks, suggesting that algorithmic shaping operates within user value judgments that are not fully malleable. Finally, multivariable models visualize the platform indicator as the dominant predictor across outcomes, but with modest explained variance (adjusted *R*^2^ ≤ 0.06), underscoring that creator practices and audience routines co-produce dissemination alongside ranking logics, and that much of the variance likely depends on dynamic platform conditions not captured in the present cross-sectional design.

Creator-type contrasts reinforce the interaction between filtering and practice. Douyin's interaction dynamics disproportionately benefit individual creators: KOLs and amateurs achieve higher engagement indices than their Bilibili counterparts (KOL *r* = 0.45; amateurs *r* = 0.27). Production choices signal rule learning: on Douyin, KOLs extend duration relative to amateurs (162.6 s vs. 116.2 s), balancing “enough information” with the short-video cadence to optimize recommendation exposure. In Bilibili's depth-oriented environment, amateurs lead overall output and KOLs pivot toward longer, didactic formats (instruction means 521–641 s among KOLs/athletes), indicating that creators reconfigure narrative arcs to align with completion, bookmarking, and community discussion signals. That official and athlete accounts do not enjoy platform-wide advantages (small or null effects) suggests a partial decentering of traditional institutional authority in these socio-technical ecologies.

The divergence in instantaneous interactions alongside convergence in bookmarking points to two coexisting accumulation regimes. On Douyin, users accrue *emotional capital* via rapid, low-cost signals (likes, brief comments, quick shares) that circulate spectacular moments—consistent with the platform's concentration in highlights and explanatory/analysis clips (75 highlights; 34 explanatory/analysis). On Bilibili, users accrue *knowledge capital* via time-intensive behaviors (completion, bookmarking, in-depth discussion), reflected in the heavier presence and longer duration of instructional content (63 items; 428.5 s mean). The near-identical bookmarking suggests a shared, cross-platform recognition of “enduring value” (e.g., technique breakdowns, rule explanations), marking a boundary condition for algorithmic influence: recommendation can steer *how* people engage moment-to-moment more readily than *what* they judge to be worth returning to.

The findings are also legible as two economic rationalities. Douyin manifests a *traffic economy* that rewards attention intermediation by highly skilled KOLs. Here we observe standardized, repeatable production workflows (emotion-forward titling, tight cuts, optimized lengths) that scale highlights/explanatory clips efficiently but risk homogeneity. Bilibili aligns more with a *community value* logic in which amateurs' authenticity and peer-to-peer didactics constitute central assets; longer-form instruction trades short-run reach for community learning and retention. These logics shape not only content supply but also creator–audience relations: producer → consumer conversion funnels on Douyin vs. learner ↔ sharer reciprocity on Bilibili. Neither model is categorically superior; each foregrounds distinct public values—immediacy and spectacle vs. pedagogy and cohesion. These differences are likely reinforced not only by ranking logics and interface design, but also by platform-specific monetization arrangements, which make high-frequency attention capture more compatible with some creator strategies and community-based retention or knowledge-oriented production more compatible with others.

Cross-platform differences illuminate a broader transformation from *embodied practice* to *digital symbolization*. Douyin's micro-duration “spectacle grammar” compresses complex skills into visually arresting fragments—rewarding *watching* badminton as a sensory experience. Bilibili's didactic grammar translates embodied know-how into codified, replayable knowledge objects—rewarding *studying* badminton as a cumulative craft. Both modalities expand access but redistribute what counts as legitimate participation: from being on court to curating, remixing, and debating representations of play. The risk is asymmetric: attention-optimized symbolization can “de-skill” understanding if it severs spectacle from context; knowledge-optimized symbolization can over-cognize technique if it neglects felt, situational know-how. Designing bridges between the two—such as interface-supported “knowledge trails” that link a short highlight to a drill clip and then to a full tutorial—offers a more concrete path for preserving body knowledge within scalable media forms. In practice, platforms could test whether these sequenced links increase tutorial click-through, completion, bookmarking, and technique-related commenting, thereby improving the educational value of sports-health content rather than only its immediate visibility.

For platforms. A more balanced metric portfolio would temper the dominance of instantaneous counters by elevating depth signals (session-level completion, structured bookmarking, reflective comments) in ranking for technique-rich sports. Investments in recommendation diversity (e.g., interleaving highlight clips with companion instructional segments) could mitigate homogenization without sacrificing engagement. For creators. Align format with platform incentives but build *cross-format bridges*: on Douyin, pair spectacle with clear calls-to-save and pointers to long-form teaching; on Bilibili, chunk long instruction into coherent, navigable modules and surface “aha” moments to sustain momentum. For sport organizations. Blend institutional credibility with grassroots reach by partnering with KOLs and amateurs on *sequenced* content (highlight → drill → full explanation) and use bookmarking/playlist features as scaffolds for progressive learning.

Several caveats bound interpretation. First, our corpus captures a single collection window (June 2025) and platform-native discovery orders (“most likes” on Douyin; “most views”/“most bookmarks” on Bilibili). This *ecological* sampling preserves each platform's logic but entangles ranking with content exposure and may under-represent emergent creators or niche formats. Therefore, the observed differences should be interpreted as differences in content as surfaced by platform-specific ranking systems, rather than as outcomes drawn from a neutral common sampling frame. Second, outcomes rely on publicly visible counters; richer, person-level behaviors (watch time, replays, session context) would sharpen inferences about depth. Third, observational comparisons cannot isolate causal effects of algorithms from correlated creator strategies and audience composition. Fourth, sport specificity matters: badminton's combination of high technique density, learnable micro-skills, and strong dual orientation toward both participation and spectating may accentuate the contrast between highlight-driven attention and instructional engagement. The findings are therefore more readily transferable to other technique-rich, video-teachable sports, whereas sports organized more around collective tactics, event atmosphere, or spectator identity may display different platform dynamics and should be examined separately.

Future work should combine time-series designs with exogenous shocks (policy/interface changes), creator interviews, field experiments (e.g., cross-posting with randomized metadata/length), and network-aware models that incorporate diffusion structure and session-level telemetry.

## Data Availability

The data analyzed in this study were derived from publicly accessible videos on Douyin and Bilibili and from the coding records generated by the authors. The original video content is not redistributed publicly because of platform-use restrictions and third-party content considerations. The coded dataset, codebook, and related analysis materials supporting the findings of this study are available from the corresponding author upon reasonable request.
